# Subcutaneous emphysema in a case of infective sinusitis: a case report

**DOI:** 10.1186/1752-1947-4-235

**Published:** 2010-08-02

**Authors:** Rasheed Zakaria, Haris Khwaja

**Affiliations:** 1 Department of Surgery, Chelsea & Westminster NHS Foundation Trust, 369 Fulham Road, London, SW10 9NH, UK; 2 Department of Surgery, Cleveland Clinic, 9500 Euclid Avenue, Cleveland, OH 44195, USA

## Abstract

**Introduction:**

Subcutaneous emphysema with pneumomediastinum is a rare phenomenon with a high morbidity and may occur spontaneously.

**Case presentation:**

A 30-year-old Caucasian man presented with sudden onset of a painful, swollen neck and was found, via clinical and radiological examination to have subcutaneous emphysema. A swallow study showed no oesophageal perforation. Computed tomography of his neck and thorax demonstrated pneumomediastinum but no other pathology. Management was conservative with intravenous antibiotics, fluids and no oral intake. He had a history of a productive cough and a flexible nasoendoscopy found purulent sinusitis which was treated with topical nasal washes. The patient was discharged after 72 hours and will be followed up by the otolaryngology-head and neck service.

**Conclusions:**

Infective sinusitis is a rare cause of subcutaneous emphysema and pneumomediastinum. It may be managed conservatively provided there is early recognition and exclusion of more serious pathology, such as a ruptured trachea or oesophagus.

## Introduction

Subcutaneous and mediastinal emphysema is an uncommon phenomenon with a significant morbidity and mortality. It is usually secondary to infection of the mediastinum, pericardium or lung parenchyma, and is particularly associated with mechanical ventilation, soft tissue infections and underlying pathology of the trachea, oesophagus or bronchial tree. Prompt recognition with treatment of sepsis and repair of any perforated viscus, if indicated, are the main features of management. Here we describe an unusual case of a patient with a short history of seemingly spontaneous subcutaneous emphysema and pneumomediastinum. Forceful paroxysms of coughing due to a purulent sinus infection were identified as the most likely cause. The patient did not require operative intervention and fully recovered with prompt investigation and conservative treatment.

## Case presentation

A 30-year-old Caucasian man presented to the general surgical service at our institution complaining of pain and skin swelling over his chest for the last 12 to 24 hours. He gave a one-week history of having had an upper respiratory tract infection with purulent nasal discharge and frequent, forceful coughing episodes. He felt hot and sweaty but otherwise systemically well, with no history of any other medical problems or regular medications. On physical examination he was diagnosed with subcutaneous surgical emphysema bilaterally from below the mandible to approximately three inches below the clavicles. He had no cervical lymphadenopathy. Abdominal and cardiac examinations were unremarkable. He had normal oxygen saturation in room air and his chest was clear.

**Figure 1:**
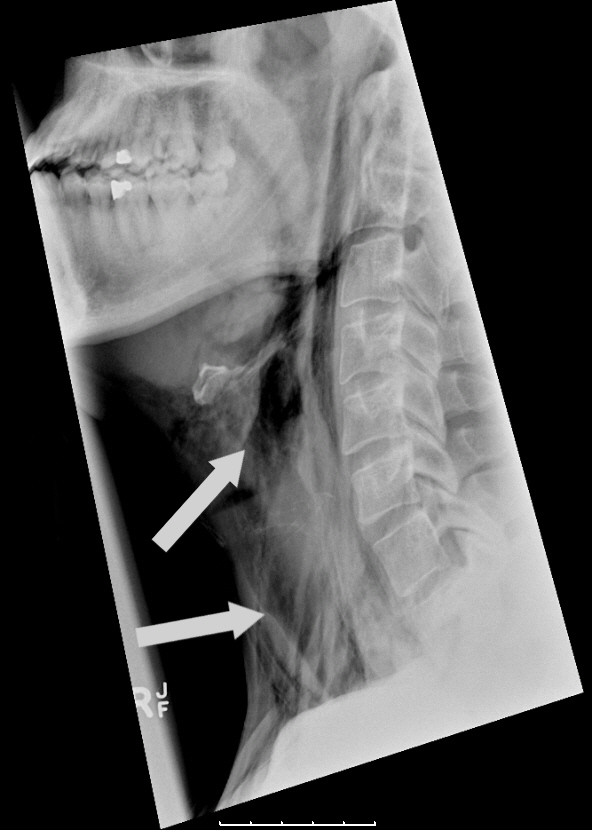
**Lateral X-ray of neck showing subcutaneous emphysema**.

Laboratory tests showed a white cell count of 12.2 × 10^9^/L (normal range 4 to 10 × 10^9^/L), C-reactive protein was 8 g/dL (normal range < 5 g/dL) with urea, creatinine, sodium, potassium and liver enzymes all within normal limits. An ECG showed sinus rhythm with no tachycardia. Plain radiographs of his neck and chest, taken in the emergency department, demonstrated marked surgical emphysema with pneumomediastinum, as shown in Figure 1. There was no free air under his diaphragm.

**Figure 2:**
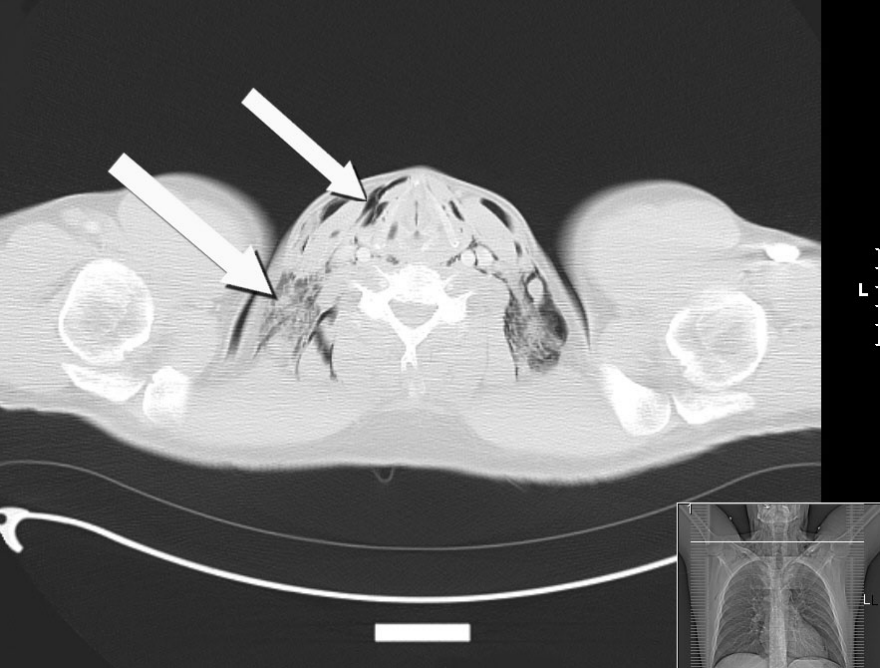
**Axial section CT neck/thorax showing subcutaneous emphysema and pneumomediastinum**.

He was admitted overnight and kept nil by mouth pending further investigation. He was given IV normal saline 1 litre every eight hours and started on IV antibiotics: cefuroxime (1.5 g three times per day) and metronidazole (500 mg three times per day). A water soluble contrast (Gastrograffin) swallow was performed the next day to detect a possible oesophageal perforation; however, no leak was found. A computed tomography (CT) scan of his neck and chest taken later that day showed extensive surgical emphysema in the pre-vertebral and pre-tracheal compartments of the neck in communication with pneumomediastinum, but the scan did not identify a perforated viscus or any fluid collections. Slices of this study are shown in Figure 2.

On day three he remained systemically well and was afebrile. An urgent review by the otolaryngology-head and neck surgeons was requested. Flexible nasoendoscopy showed bilateral infective sinusitis with thick post-nasal drip and a haemorrhagic vocal cord on the right with no associated pathological lesions seen. Nasal cleaning with saline washes was initiated four times daily along with topical steroid nasal spray twice daily. He tolerated sips of water followed by a build-up to solid food over the following 24 hours. He was discharged after a further 48 hours with two weeks of equivalent oral antibiotics (co-amoxiclav 625mg three times daily) and continued nasal washes. He will be followed up by the otolaryngology-head and neck service.

## Discussion

Subcutaneous emphysema and pneumomediastinum is most often seen in association with blunt or penetrating trauma, soft-tissue infections, or any condition that creates a gradient between intra-luminal and extra-luminal pressures [1]. The case we report here is rare in that our patient was systemically well with only a short history of cough. In the absence of any signs of soft tissue infection, pulmonary disease or trauma in a patient with no relevant medical history, perforation of a cervical viscus was rightly suspected. Recognition of this condition may be difficult. Our patient presented with chest pain and subcutaneous emphysema. These are the most common symptoms of a perforated cervical viscus along with shortness of breath [2]. A chest X-ray identified pneumomediastinum in this case but this is not a universally sensitive investigation [3].

The first cause for subcutaneous emphysema considered by the admitting team was Boerhaave's syndrome. First described in the 18^th ^century, this is a transmural perforation of the esophagus caused by a sudden rise in intramural pressure during forceful emesis. The patient demonstrated signs and symptoms consistent with this; Mackler's triad - comprising vomiting, subcutaneous emphysema and chest pain - is said to be diagnostic for spontaneous esophageal rupture though it may rarely be present [4]. Lateral neck film for cervical perforation and upright AP chest film for thoracic perforations may show air in the prevertebral and pretracheal fascial spaces. The negative swallow study in our patient suggested there was no esophageal perforation, though these are positive in some 90% of cases. Nevertheless, it should be noted that water soluble contrast media, as used in this case, are less likely to extravasate and therefore less likely to detect a leak than barium-based media [5]. Though there are cases of spontaneous esophageal rupture without an antecedent history of vomiting, these appear to have involved an already weakened esophagus due to some other disease process such as mural infection or malignancy [6]. Hence, there may have been little value in a swallow study in a patient presenting like this with subcutaneous emphysema, and CT may be a more useful first line investigation after a regular X-ray[7].

Following a negative swallow study our patient promptly went on to have a CT scan of the neck and thorax to rule out tracheal rupture. This is a common cause of subcutaneous emphysema above the clavicles but is most often due to trauma [8] or iatrogenic injury during difficult intubation [9] neither of which applied to our patient. Relevant to our case, tracheal rupture has also been reported in cases of forceful coughing, for example due to upper respiratory tract infection. However, besides pediatric patients [10] the only reported adult cases have had considerably weakened soft tissues due to tracheobronchomalacia [11] or long term corticosteroid use [12].

Endoscopic examination is not mandatory but in this case yielded the diagnosis of infective sinusitis while the finding of a haemorrhagic vocal cord would favor a subglottic site for tracheal rupture. With regard to mediastinal injury, a CT thorax scan excluded any hilar injury or intrathoracic tracheal rupture in this case. Alveolar rupture due to expiration against a closed airway may lead to pneumomediastinum and subsequently subcutaneous emphysema as air tracks up along the hila. This has been repeatedly described in asthma, although more so in adolescent and paediatric patients [13] but is also reported in women during labor. More recently there has been an increased incidence of this strongly associated with cocaine use, though the mechanism is unclear [14]. Finally some 20% of cases will remain truly idiopathic [3].

Management was conservative in this instance and similar cases report favorable outcomes from antibiotics, fluids and observation although rarely mediastinal shift or fluid collection mandates surgical exploration or chest tube placement [15]. We could have taken serial radiographs to ensure air was being resorbed, though daily clinical review was a reasonable alternative strategy.

## Conclusions

Subcutaneous emphysema of the chest wall or neck presenting with or without chest pain and shortness of breath is a rare entity. The condition needs prompt recognition and a careful history and examination to establish the possible causes and sequelae. Plain radiographs and ultimately CT of the neck and thorax are needed to establish if there is underlying pneumomediastinum and to exclude fluid collections in the lung, pericardium or mediastinum which may need drainage percutaneously or surgically. Important causes of pneumomediastinum and subcutaneous emphysema are tracheal or oesophageal rupture (the so-called Boerhaave's syndrome). Endoscopic examination and swallow studies may assist in making such diagnoses. Purulent sinusitis causing a violent cough is one possible cause of spontaneous pneumomediastinum in an otherwise healthy individual. Conservative management with fluid and antibiotics may be appropriate but close observation is necessary for signs of sepsis or respiratory compromise.

## Abbreviations

CT: computed tomography; ECG: electrocardiogram; IV: intravenous; WBC: white blood cell count.

## Consent

Informed consent was obtained from the patient for publication of this case report and accompanying images. A copy of the written consent is available for review by the Editor-in-Chief of this journal.

## Competing interests

The authors declare that they have no competing interests.

## Authors' contributions

RZ wrote the description of the case, HK and RZ drafted the literature review. All authors have read and approved the final manuscript.
